# *Siraitia grosvenorii* Residual Extract Attenuates Atopic Dermatitis by Regulating Immune Dysfunction and Skin Barrier Abnormality

**DOI:** 10.3390/nu12123638

**Published:** 2020-11-26

**Authors:** Yoon-Young Sung, Heung-Joo Yuk, Won-Kyung Yang, Seung-Hyung Kim, Dong-Seon Kim

**Affiliations:** 1 Herbal Medicine Research Division, Korea Institute of Oriental Medicine, 1672 Yuseongdae-ro, Yuseong-gu, Daejeon 34054, Korea; yysung@kiom.re.kr (Y.-Y.S.); yukhj@kiom.re.kr (H.-J.Y.); 2 Institute of Traditional Medicine and Bioscience, Daejeon University, 62 Daehak-ro, Dong-gu, Daejeon 34520, Korea; ywks1220@dju.kr (W.-K.Y.); sksh518@dju.kr (S.-H.K.)

**Keywords:** allergic inflammation, filaggrin, keratinocytes, nahangwa, skin barrier

## Abstract

Atopic dermatitis is a persistent inflammatory skin disorder. *Siraitia grosvenorii* fruits (monk fruit or nahangwa in Korean, NHG) are used as a natural sweetener and as a traditional medicine for the treatment of asthma and bronchitis. We evaluated the activity of *S. grosvenorii* residual extract (NHGR) on allergic inflammation of atopic dermatitis in a *Dermatophagoides farinae* mite antigen extract (DfE)-treated NC/Nga murine model and in vitro. Oral administration of NHGR significantly reduced epidermal hyperplasia and inflammatory cell infiltration in the skin lesions of DfE-induced atopic dermatitis, as well as the dermatitis severity score. NHGR reduced serum immunoglobulin E levels. Splenic concentrations of IFN-γ, interleukin (IL)-4, IL-5, and IL-13 were reduced by NHGR administration. Immunohistofluorescence staining showed that NHGR administration increased the protein levels of claudin-1, SIRT1, and filaggrin in atopic dermatitis skin lesions. In addition, NHGR inhibited the phosphorylation of mitogen-activated protein kinases and decreased filaggrin and chemokine protein expression in TNF-α/IFN-γ-induced human keratinocytes. Moreover, NHGR also inhibited histamine in mast cells. The quantitative analysis of NHGR revealed the presence of grosvenorine, kaempferitrin, and mogrosides. These results demonstrate that NHGR may be an efficient therapeutic agent for the treatment of atopic dermatitis.

## 1. Introduction

Atopic dermatitis (AD) is a persistent inflammatory skin disorder that is characterized by acute itching and relapsing eczematous lesions with eosinophilia and increased immunoglobulin (Ig) E in serum [1]. Patients with AD have an increased hazard for allergic diseases such as allergic rhinitis, asthma, and food allergies (the so-called atopic march), as well as other immune-related inflammatory disorders such as psoriasis, inflammatory bowel disease, and mental health disorders [2]. Both immune system dysregulation and cutaneous epidermal skin barrier impaired functioning are involved in the progression of AD, although the exact mechanisms are still unclear [3]. Mutational loss and reduced expression of epidermal proteins such as filaggrin (filament-aggregating protein) affect diverse pathways that are relevant to epidermal skin barrier dysfunction, including altered lipid formation, unbalanced keratinocyte differentiation, impaired tight-junction formation, water loss, and stratum corneum acidification. Further, filaggrin deficiency increased exposure to allergens, with subsequent immune B cell and T cell priming, and released innate immune Th2-promoting cytokines and chemokines from keratinocytes [4,5]. Tight junctions are major layers to the skin barrier, and claudin-1, a main factor of tight junction in the epidermis, plays an important role in skin barrier function, epithelia permeability, and transepidermal water loss [6]. Claudin-1 is reduced in dermal lesions of patients with AD, triggering skin inflammation [7].

Type 2 immune cytokines such as IL-5, IL-4, IL-13, and IL-31 play important roles in skin barrier dysfunction, chemokine production, suppression of antimicrobial peptides, and allergic inflammation in AD [8]. Th2 cells are associated with the early stage of AD. On the other hand, Th1 cells, which produce interferon (IFN)-γ and IL-12, contribute to the development of the late stage of AD [9,10]. Mast cell degranulation plays a major function in the onset of allergic disorders through differential regulation of T lymphocytes. Histamine regulates the effective balance between Th1 and Th2 cells by supporting a Th2 shift [11]. Histamine contributes to the advance of allergic inflammations by enhancing the production of pro-inflammatory cytokines, such as IL-1β, IL-6, and IL-1α, and chemokines such as regulated on activation T cell expressed and secreted (RANTES) in local tissues [12].

*Siraitia grosvenorii* (Swingle) fruits (monk fruit or nahangwa in Korean, NHG), a member of the family Cucurbitaceae, are known as a natural sweetener and as a traditional herbal medicine to treat bronchitis, asthma, cough, and sore throat [13]. *S. grosvenorii* fruit is extracted with water and then its residue is generally discarded. Previous reports have shown that *S. grosvenorii* extract and its components have various pharmacological properties, such as anti-tussive, anti-inflammatory, immunologic, hypoglycemic, anti-carcinogenic, and anti-oxidative activities [14]. Although *S. grosvenorii* residual extract (NHGR) is an original material available at a low cost, no studies have evaluated the anti-allergic activities of NHGR. Therefore, our work investigated the anti-allergic effects of NHGR on mite allergen-induced skin inflammation as a representative of the in vivo model for AD, as well as immune cells and keratinocytes.

## 2. Materials and Methods

### 2.1. Sample Supplement

Dried *Siraitia grosvenorii* fruits were gained from Hunan Huacheng Biotech, Inc. (Changsha, Hunan, China). The fruits were washed with clean water and were extracted with water. After water extraction, the residues were collected and the dried residue was extracted three times with 70% ethanol. The residual extracts were filtered by a high-velocity centrifugal machine, concentrated by a vacuum system, sterilized under ultra-high-temperature, dried at 40 °C in a microwave for 1–2 h, and stored at 4 °C. The extract was kept in the herbarium of the Herbal Medicine Research Division at the Korean Institute of Oriental Medicine.

### 2.2. Ultra-Performance Liquid Chromatography-Quadrupole Time-of-Fight Mass Spectrometry (UPLC–QTOF–MS) Analysis

The *Siraitia grosvenorii* residual extract (NHGR) samples were analyzed using an ultra-performance liquid chromatography (UPLC) system (Waters Corporation, Milford, MA, USA) with an autosampler and a detector coupled to a Waters QTof Premier^TM^ mass spectrometer. The samples were separated on an ACQUITY BEH (1.7 µm, 2.1 × 100 mm) C18 column at a flow rate (0.4 mL/min). All analytes were eluted by water-acetonitrile (A-B) mobile system containing 0.1% formic acid. The linear gradient was conducted as follows: 0 min, 15% B; 0–1 min, 15% B; 1–9 min, 15–40% B; 9–11 min, 40–90% B; 11–11.3 min, 90–100% B; 11.3–13.1 min, 100% B; and 13.1–13.2 min, back to 15% B. The mass spectrometer was manipulated in negative ion mode under the following controls: Cone voltage 40 V, source temperature 110 °C, capillary voltage 2.5 kV, and desolvation temperature 350 °C. A leucine–enkephalin ([M − H]^−^m/z 554.2615) reference solution was utilized as the lock mass. Five main standards (1: Grosvenorine, 2: Kaempferitrin, 3: Mogroside V, 4: Mogroside IV, 5: Mogroside III) for analysis were purchased from ChemFaces (Wuhan, Hubei, China).

### 2.3. Animal Testing

NC/Nga mice (male, 7 weeks old) were supplied by Central Lab Animal Inc. (Seoul, Korea). The animals were adapted to their habitat for one week. The animal study was approved by Daejeon University’s Animal Care and Use Committee (Ethical approval code. DJUARB2019-042), and animal experiments were performed in accordance to the Guide for Care and Use of Laboratory Animals and the guidelines regarding animal care and use (National Research Council of USA, 1996).

**Figure 1:**
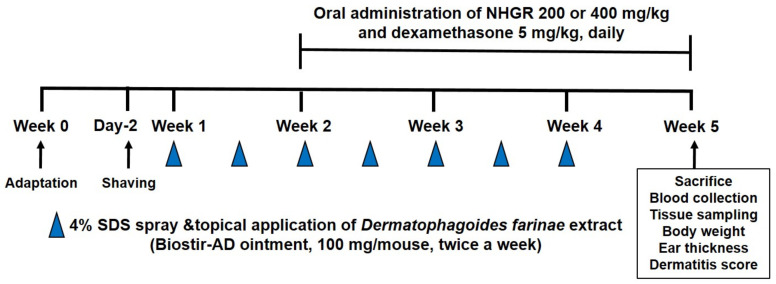
Experimental design for induction of atopic dermatitis and application with the *Siraitia grosvenorii* residual extract (NHGR) in mice.

The experimental design is shown in Figure 1. Induction of AD-like skin lesions in animals were performed as previously reported [15]. The fur on the back of each mouse was shaved and 4% sodium dodecyl sulfate (150 μL) was sprayed to the shaven back skins for the disruption of the skin barrier. After 4 h, AD was induced via topical application of 100 mg of the house-dust mite *Dermatophagoides farinae* extract (DfE) ointment (Biostir, Hyogo, Japan) to the skin twice a week for three weeks. The animals with DfE-applied AD were split into the following four groups (*n* = 6): DfE-applied mice (DfE), DfE application and dexamethasone 5 mg/kg administration (Dexa 5 mg/kg) group, DfE application and NHGR administration (NHGR 200 mg/kg) group, and DfE application and NHGR administration (NHGR 400 mg/kg) group. The normal control (NC) group was the only vehicle without DfE application. Dexamethasone and NHGR were dissolved in a vehicle (saline). The same volume of vehicle was treated to the normal and DfE control mice. Ear thickness was assessed with a micrometer (Mitutoyo, Kanagawa, Japan). The severity and the clinical score of dermatitis were assessed as previously reported [16].

### 2.4. Analysis of White Blood Cells from Peripheral Blood

Blood samples were obtained from the animals by cardiac puncture and white blood cells such as leukocytes, basophils, neutrophils, and eosinophils were analyzed. The total cell numbers were analyzed using a CELL-DYN^®^ 3200 hematology analyzer (Abbott, Santa Clara, CA, USA).

### 2.5. Splenocyte Culture

After spleen isolation, red blood cells in single-cell suspensions were eliminated by a red blood cell lysis solution. The spleen cells were cultured in the stimulation or no-stimulation of an anti-CD3 (0.5 μg/mL) (eBioscience, San Diego, CA, USA). After incubation for 24 h, the cell supernatant was gathered to measure the concentrations of IFN-γ, IL-13, IL-4, and IL-5.

### 2.6. Enzyme-Linked Immunosorbent Assay

Enzyme-Linked Immunosorbent Assay (ELISA) kits were used according to the manuals for the measurement of total IgE concentrations in serum (Oxford Biomedical Res., Oxford, MI, USA) and IFN-γ, IL-4, IL-5, and IL-13 concentrations in the supernatant of cultured splenocytes (R&D, St. Louis, MO, USA).

### 2.7. Histological Examination

The back skin of the animals was removed, embedded in paraffin, and cut to a thickness of 4 μm. The histological examination of the paraffin-sections for evaluating the degree of inflammation was evaluated by toluidine blue (TB) staining and hematoxylin and eosin (H&E) staining.

### 2.8. Immunofluorescence Staining

The sections were blocked in 5% bovine serum albumin at room temperature for 1 h. The nucleus was stained with Hoechst 33342 solution for 20 min. The primary antibody (1:50 dilution) containing filaggrin, Sirtuin 1 (SIRT1), and Claudin-1 (Abcam, Cambridge, MA, USA) was treated, followed by overnight incubation at 4 °C, and then a secondary antibody was added for 4 h. The stained slides were mounted and imaged with a confocal microscope (Nikon, Tokyo, Japan).

### 2.9. Histamine Release in MC/9 Mast Cells

The MC/9 mouse mast cells were gained from the American Type Culture Collection (Manassas, VA, USA). The cells were cultured in Dulbecco’s modified Eagle’s medium (DMEM) supplemented with 0.05 mM 2-mercaptoethanol (Sigma, Seoul, Korea), 10% rat T-STIM (Becton Dickinson, Franklin Lakes, NJ, USA), 2 mM L-glutamine, and 10% fetal bovine serum (FBS). The viability from the cells was evaluated using a Cell Counting Kit-8 (BioVision, Milpitas, CA, USA). Histamine concentrations in the cell supernatant were measured using a histamine immunoassay kit (Oxford Biomedical Research, Oxford, MI, USA). The cells were pretreated with various concentration of extracts before 30 min. Then, compound 48/80 (25 µg/mL) was treated and the cells were incubated for an additional 30 min. After the reaction was stopped, the optical density was measured at 650 nm using a microplate reader (BioRad, Hercules, CA, USA).

### 2.10. Chemokine Assay in HaCaT Keratinocyte Cells

The HaCaT immortalized keratinocytes were maintained in DMEM supplemented with 100 U/mL penicillin, 100 μg/mL streptomycin, and 10% FBS at 37 °C. For measurement of chemokines, the cells (1 × 10^6^ cells/well) were seeded into each well of 6-well plates and treated with various concentrations of NHGR. After pre-incubation for 1 h, the cells were co-treated with TNF-α (10 ng/mL) and IFN-γ (10 ng/mL) for 24 h. As a positive control, dexamethasone (1 μM) was used. The secretion levels of RANTES, TARC, and thymic stromal lymphopoietin (TSLP) in the cell supernatant was determined using ELISA kits, respectively (R&D Systems, Minneapolis, MN, USA). Absorbance was determined at 450 nm.

The viability from the cells was determined by a 3-(4,5-dimethylthiazol-2-yl)-2,5-diphenyltetrazolium bromide (MTT) solution. The cells (5 × 10^4^ cells/well) were seeded into each well of 96-well plates and were treated with various concentrations of NHGR for 24 h. Then, a MTT (100 μL) was inserted to each well. After reaction for 3 h, dimethyl sulfoxide was included to solubilize the purple formazan. The absorbance was evaluated at 540 nm.

### 2.11. Western Blotting

HaCaT cells (1 × 10^6^ cells/well) in a 6-well plate were incubated with DMEM medium alone or with TNF-α/IFN-γ in the presence or absence of NHGR for 15 min for the detection of mitogen-activated protein kinases (MAPKs) or for 24 h for the expression of filaggrin. β-actin (Cell Signaling Tehnology, Beverly, MA, USA) was used as an internal control. Protein extraction from cell lysates was performed using PRO-PREP extraction buffer (Intron, Seoul, Korea). Proteins (10 μg) were separated by a 4–12% gradient sodium dodecyl sulfate-polyacrylamide gel and were transferred onto membranes using a trans-blot Turbo transfer system (Bio-Rad, Hercules, CA, USA). Membranes were blocked with EzBlock Chemi solution (ATTO, Kyoto, Japan) and placed at 4 °C overnight using primary antibodies against filaggrin, p38, p-p38, extracellular signal-regulated kinase (ERK), p-ERK, c-Jun N-terminal kinase (JNK), and p-JNK, all of which were purchased from Santa Cruz Biotechnology (Santa Cruz, CA, USA). The membranes were then placed with the secondary antibodies (Cell Signaling) for 1 h. Membranes were reacted with an ECL detection reagent (Amersham Bioscience, Buckinghamshire, UK), and protein bands were visualized using LAS-4000 (Fujifilm, Tokyo, Japan).

### 2.12. Statistical Analysis

Result are presented as the mean ± standard errors of the mean (SEM). Significant differences were regarded significant at *p* < 0.05. One-way analysis of variance (ANOVA) and a multiple comparison Dunnett’s test were conducted using Prism 7.0 (GraphPad Software Inc., San Diego, CA, USA).

## 3. Results

### 3.1. Identification of Main Compounds in NHGR by UPLC–QTOF–MS

**Figure 2:**
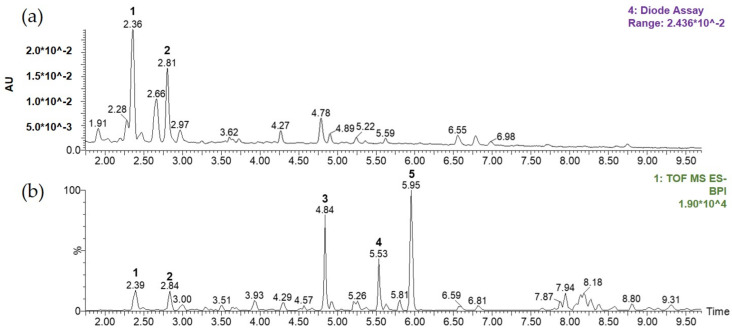
Representative chromatograms of the 70% ethanol extract from the *Siraitia grosvenorii* residual extract (NHGR): (**a**) Photodiode array (PDA) chromatogram and (**b**) total ion current-base peak intensity (TIC–BPI) chromatogram.

Figure 2 shows that complete chromatographic separation of the major compounds within NHGR was achieved within 10 min of monitoring elution using a total ion current-base peak intensity (TIC-BPI) chromatogram and Photodiode array (PDA) detection. Five major compounds in the extracts were determined by comparison of their retention times (tR) values, UV/Vis spectra, and HR MS/MS spectra (fragmentation pattern), with the spectra of known standards and comparison with published literature [17,18]. From this information, peaks 1–5 were assigned to grosvenorine (1), kaempferitrin (2), mogroside V (3), mogroside IV (4), and mogroside III (5).

### 3.2. Effects of NHGR on DfE-Induced AD Mice

**Figure 3:**
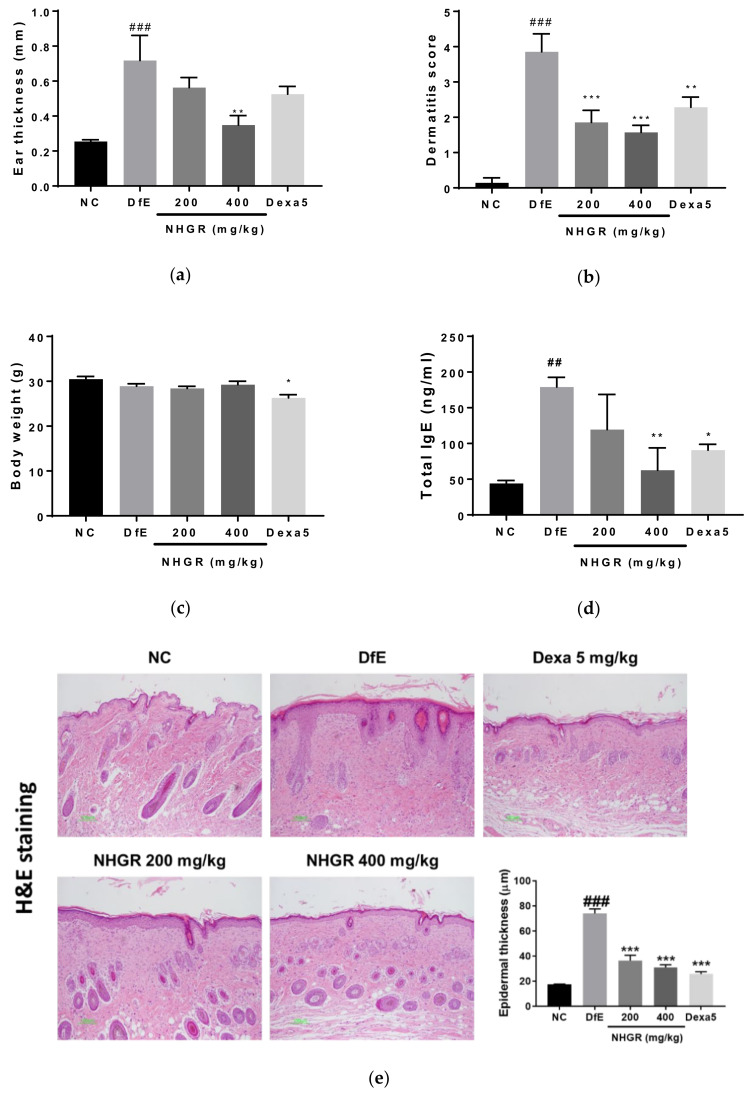

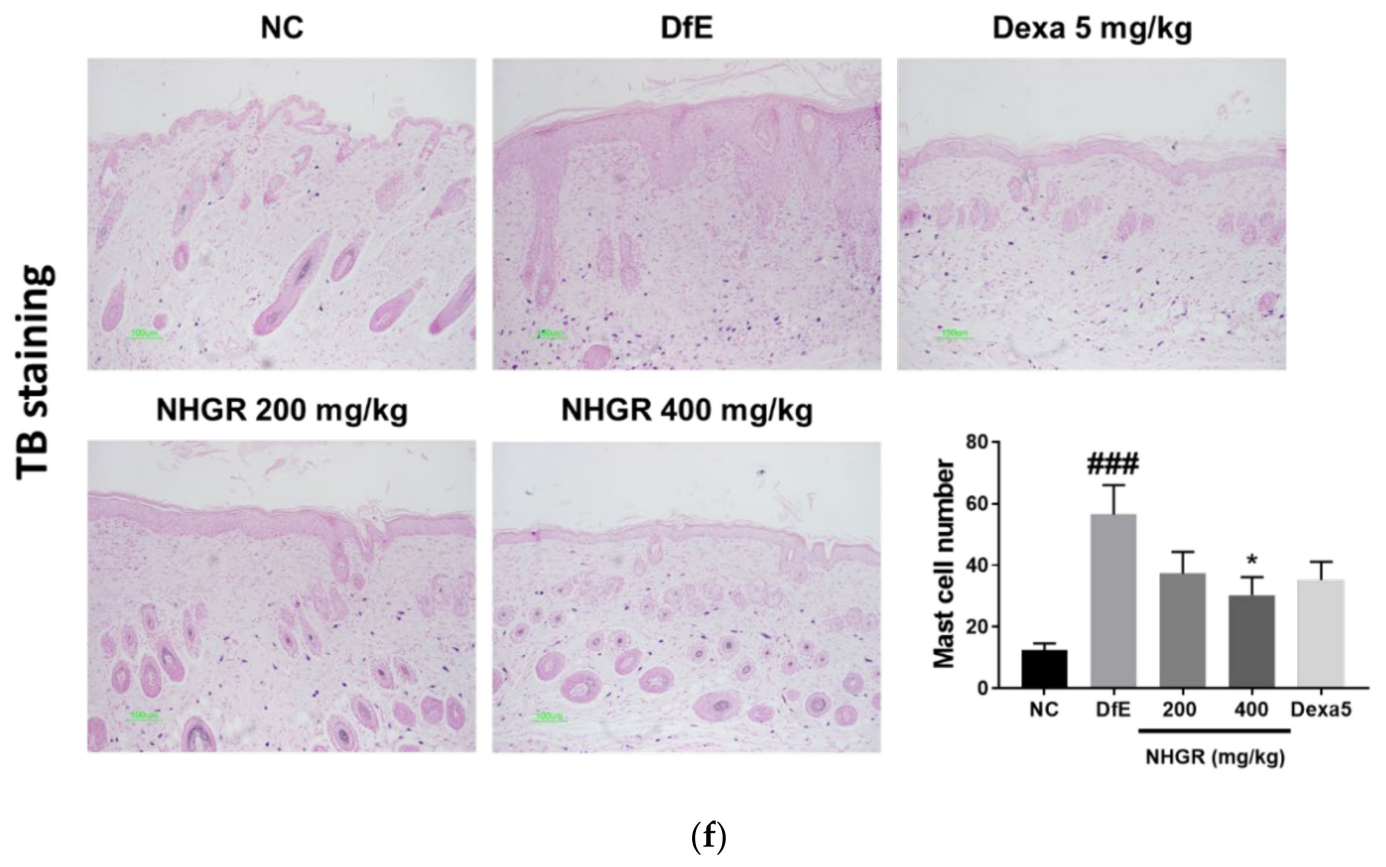
The effects of *Siraitia grosvenorii* residual extract (NHGR) on the symptoms of atopic dermatitis in mice. (**a**) Ear thickness, (**b**) dermatitis score, (**c**) body weight, and (**d**) serum total IgE concentration. Dorsal skin sections were stained with (**e**) hematoxylin and eosin as well as (**f**) toluidine blue. Epidermal thickness and number of mast cells in the stained sections were evaluated. TB, toluidine blue stain; H&E, hematoxylin-eosin stain. Results are expressed as means ± SEM (*n* = 7). ^##^ *p* < 0.01 and ^###^ *p* < 0.001 compared with NC; * *p* < 0.05, ** *p* < 0.01, and *** *p* < 0.001 compared with *Dermatophagoides farinae* extract (DfE).

To investigate the activity of NHGR oral administration on AD symptoms, dermatitis score, ear thickness, body weight, and serum total IgE concentrations were evaluated in DfE-applied NC/Nga mice. Application of DfE for three weeks caused AD symptoms such as erythema, dryness, erosion, scarring, edema, excoriation, and hemorrhage, and these dermatitis symptoms were relieved by oral administration with NHGR. The dermatitis severity score and ear swelling increased in the DfE-induced AD animals and were reduced by oral administration with 200 or 400 mg/kg NHGR and dexamethasone (Figure 3a,b). Body weight did not change in response to NHGR treatment but decreased after oral treatment with dexamethasone (Figure 3c). Circulating IgE levels as a biomarker of AD were elevated in the serum of DfE-induced mice and were significantly ameliorated by treatment with 400 mg/kg NHGR or dexamethasone (Figure 3d). In a histopathological examination using H&E and TB staining of skin lesions, DfE application to the skin lesions induced the thickening of the epidermis and the infiltration of inflammatory cells containing mast cells recovered from the NHGR treatment (Figure 3e,f).

Alteration in the immune cell types exhibit during skin inflammation, particularly eosinophils, supply critical data about the onset of AD. Hematological analysis of white blood cells showed that the total number of neutrophils and eosinophils increased in the peripheral blood of DfE-induced mice and were significantly reduced by oral administration with 200 and 400 mg/kg of NHGR (Table 1). Blood eosinophilia is show in most patients of AD, correlating nearly with severity [19].

### 3.3. Effects of NHGR on the Secretion of Th1 Cytokines and Th2 Cytokines by Cultured Splenocytes

**Figure 4:**
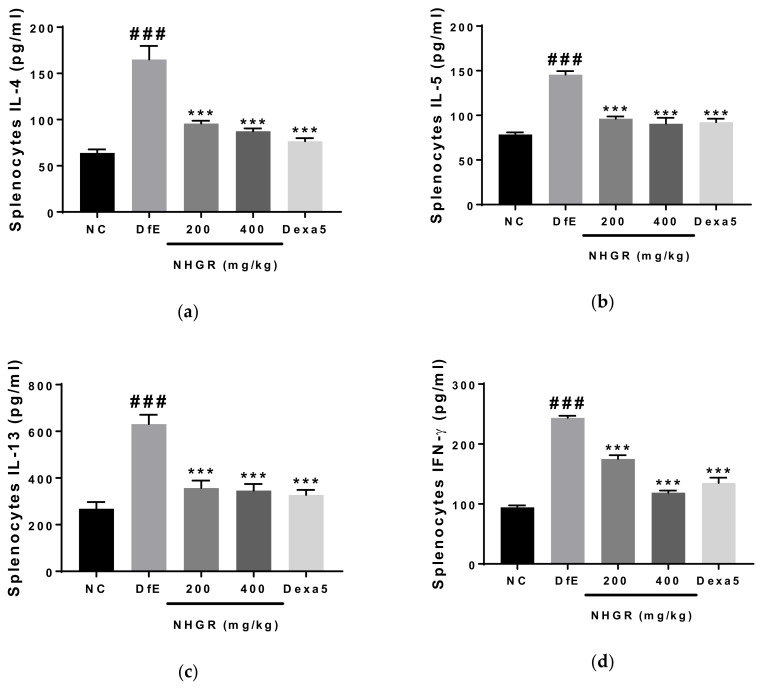
The effects of *Siraitia grosvenorii* residual extract (NHGR) on the secretion of Th1 and Th2 cytokines by cultured splenocytes. The concentrations of (**a**) IL-4, (**b**) IL-5, (**c**) IL-13, and (**d**) IFN-γ in the cell culture supernatant were determined. The cells were maintained for 48 h at a density of 1 × 10^5^ cells/well in 96-well plates using anti-CD3 antibody-coating. Data is showed as the means ± SEM (*n* = 7). ^###^ *p* < 0.001 compared with NC; *** *p* < 0.001 compared with *Dermatophagoides farinae* extract (DfE)-treated atopic dermatitis mice.

To investigate whether NHGR influences cytokine secretion in the spleen cells, IFN-γ, IL-4, IL-5, and IL-13 levels in the splenocytes were determined after the experiment. The oral administration of 200 and 400 mg/kg NHGR restored the increase of Th1 cytokine IFN-γ and Th2 cytokines (IL-4, IL-5, and IL-13) by DfE-application in the supernatant of splenocyte cultures (Figure 4). These results show that NHGR can improve AD by regulating Th2 and Th1 cytokines.

### 3.4. Effects of NHGR on the Expression of SIRT1, Claudin 1, Filaggrin in Dermal Skin

**Figure 5:**
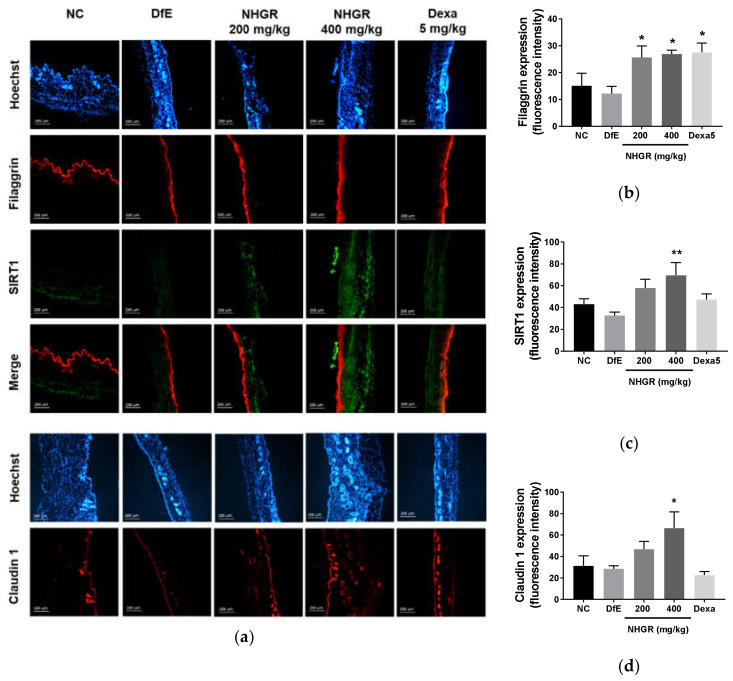
Immunohistofluorescence staining of SIRT1, claudin 1, and filaggrin in the dorsal skin tissue of NC/Nga mice. (**a**) SIRT1 (green), filaggrin (red), merge of filaggrin and SIRT1 (red), claudin1 (red), and Hoechst (blue). Densitometric quantification of filaggrin (**b**), SIRT1 (**c**), and Claudin1 (**d**). Fluorescence values quantified by the Image J program are represented as bars. Values are expressed as means ± SEM (*n* = 7). * *p* < 0.05 and ** *p* < 0.01 compared with *Dermatophagoides farinae extract* (DfE)-treated atopic dermatitis mice.

To evaluate the activity of NHGR on skin barrier function, the expression of various skin barrier proteins was investigated. The oral administration of NHGR enhanced the protein expression of filaggrin and SIRT1, the skin barrier proteins of the epidermis (Figure 5a,b). Filaggrin expression was significantly increased by an oral administration with 200 mg/kg NHGR. The 400 mg/kg NHGR treatment also increased the protein expression of Claudin-1, a tight junction protein of the epidermis (Figure 4c). These results suggest that NHGR may improve AD by regulating skin barrier dysfunction.

### 3.5. Effects of NHGR on Histamine Release from Mast Cells

**Figure 6:**
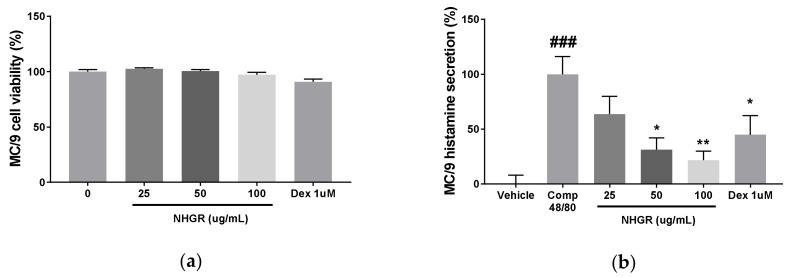
Effect of *Siraitia grosvenorii* residual extract (NHGR) on histamine secretion from MC/9 mast cells. (**a**) Cell viability and (**b**) histamine secretion. MC/9 cells (2 × 10^5^ cells/mL) were treated with NHGR (25–100 µg/mL) and compound 48/80 (25 µg/mL) for 30 min. Data is showed as means ± SEM (*n* = 3). ^###^ *p* < 0.001 versus vehicle; * *p* < 0.05 and ** *p* < 0.01 versus compound 48/80-treated control.

Using the secretion of histamine by mast cell degranulation as an allergic indicator, the anti-allergic effect of NHGR was determined on compound 48/80-induced histamine release from MC/9 mouse mast cells. NHGR did not affect cell viability and restored the compound 48/80-induced histamine secretion in a dose-dependent manner (Figure 6a,b). The treatment with dexamethasone 1 μM as a positive control inhibited histamine release.

### 3.6. Activities of NHGR on the Protein Expression of Filaggrin and Chemokine from Keratinocytes

**Figure 7:**
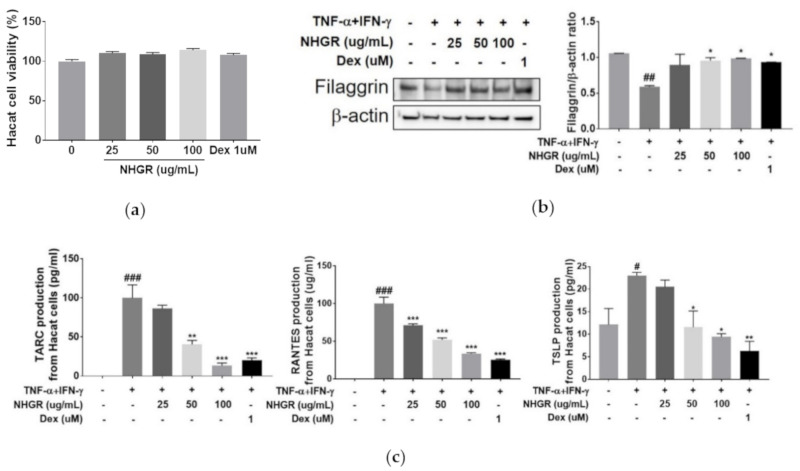

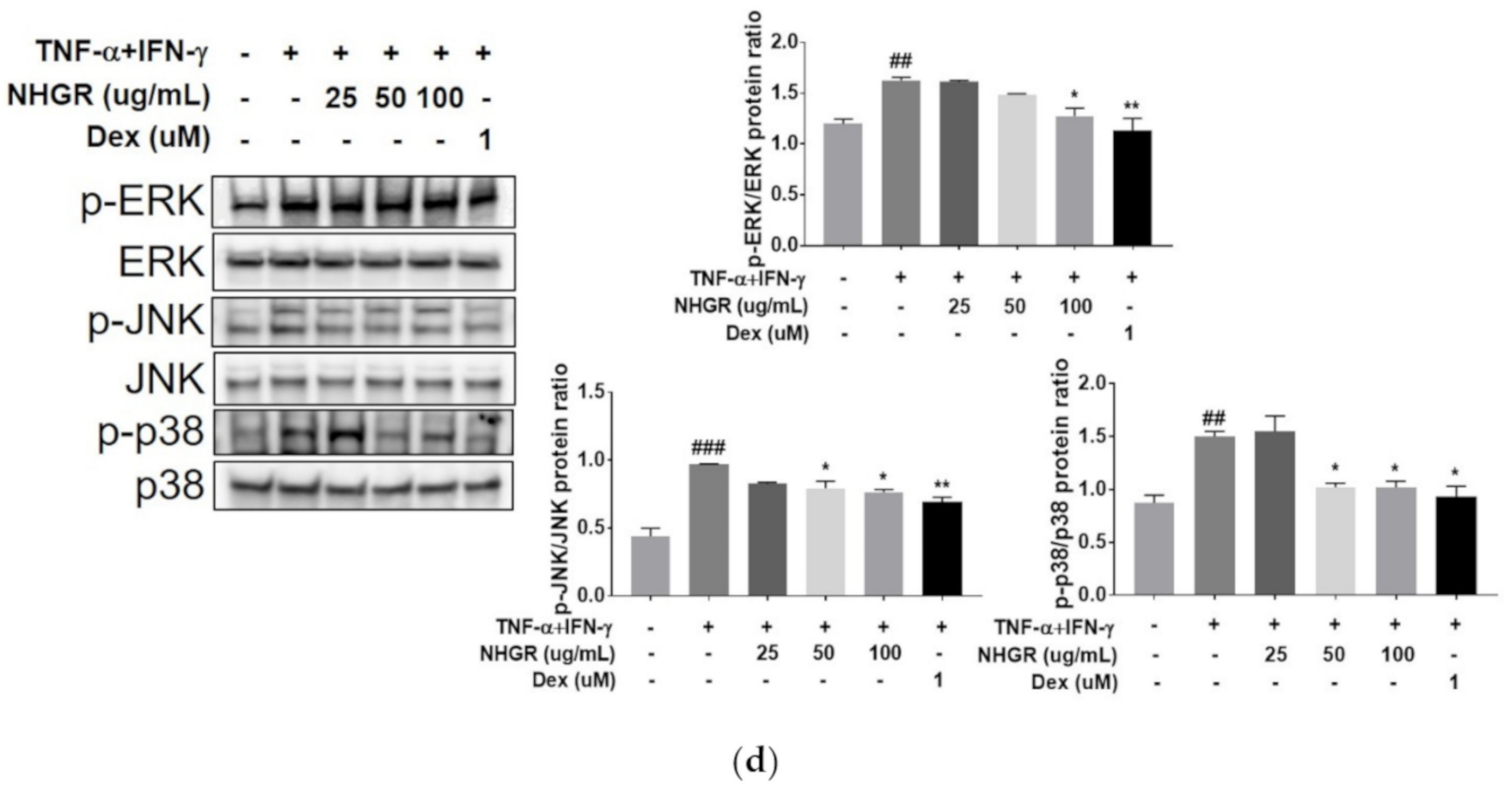
Effect of *Siraitia grosvenorii* residual extract (NHGR) on the protein expression of filaggrin and chemokine and activation of ERK, JNK, and p38 MAPKs from TNF-α/IFN-γ-induced HaCaT cells. (**a**) Cell viability, (**b**) filaggrin expression, (**c**) chemokine (RANTES, TARC, and TSLP) production, and (**d**) phosphorylation of ERK, JNK, and p38 MAPKs. Expression of protein and phosphorylation of MAPKs were examined. Results are showed as means ± SEM (*n* = 3). ^#^ *p* < 0.05, ^##^ *p* < 0.01 and ^###^ *p* < 0.001 versus vehicle; * *p* < 0.05, ** *p* < 0.01 and *** *p* < 0.001 versus TNF-α/TNF-γ-treated control.

Stimulation with both TNF-α and IFN-γ in keratinocytes led to an increased expression of various proinflammatory cytokines and chemokines. These cytokines contributed to the infiltration of inflammatory cells to dermal skin [20]. NHGR did not affect cell viability in HaCaT cells (Figure 7a). NHGR dose-dependently restored the decreased filaggrin protein expression and increased chemokine (TARC, RANTES, and TSLP) production in TNF-α and IFN-γ-treated cells (Figure 7b,c).

The expression of inflammatory factors, such as TARC and RANTES, in HaCaT human keratinocytes treated with TNF-α/IFN-γ is activated by intracellular MAPK-signaling pathways [21]. Therefore, to investigate its mechanisms of the anti-inflammatory activities of NHGR, the activities of NHGR on the phosphorylation of MAPK (JNK, ERK, and p38) were studied. The results showed that NHGR reversed ERK, JNK, and p38 phosphorylation (Figure 7d). The results demonstrated that the anti-inflammatory activities of NHGR are associated with ERK, JNK, and p38 MAPK signaling pathways.

## 4. Discussion

Our previous study demonstrated that oral administration of NHGR in ovalbumin-induced asthmatic rodents markedly inhibited lung inflammation by down-regulating Th2 cytokines [22]. These results demonstrated the potential therapeutic activities of NHGR against allergic skin inflammation such as AD. However, little is reported about the activity of NHGR on allergic skin inflammation such as AD. Thus, we evaluated the anti-allergic and anti-inflammatory activities of NHGR using the in vivo model of AD and in vitro cells.

This study showed that oral administration of NHGR improved AD-like skin lesions induced by DfE allergen and exhibited potent inhibitory activities on allergy and inflammation. NHGR enhanced the expression of skin barrier-associated proteins, such as SIRT1, filaggrin, and claudin-1, in AD skin lesions of animals. Furthermore, the increase on filaggrin expression by pro-inflammatory cytokines was restored by NHGR treatment in vitro. These results suggested that oral administration of NHGR could ameliorate AD by increasing filaggrin, SIRT1, and claudin-1 expression, supporting the restoration of the skin barrier dysfunction.

The results of this study indicated that oral administration of NHGR was associated with improvements in the inflammatory response and skin barrier dysfunction. IL-4, IL-5, and IL-13 cytokines secreted by Th2 cells, which induce immune reactions in the early stage of AD, especially IL-4, are necessary for the IgE production through Ig class switching in B cells. Th1 cytokine IFN-γ contributes to the onset of the chronic stage of AD [23]. The protein deacetylase SIRT1 supports filaggrin expression and regulates skin barrier integrity [24]. The skin barrier dysfunction sustains antigen entrance, leading to infiltration of Th cell, which can cause skin inflammation in AD [6]. Splenic expression of these Th1 and Th2 cytokines together with serum IgE levels was reduced by oral administration of NHGR in the AD model. The protein expression of SIRT1 together with filaggrin and claudin-1 increased in response to NHGR in the skin lesions. Therefore, the recovery of the skin barrier function via oral administration with NHGR may reduce the production of Ig and cytokines in AD rodents.

TARC/CCL17 is a ligand involved in Th2 lymphocytes activation and recruitment through CC chemokine receptor 4 [25]. As an epithelium-derived cytokine, TSLP activates dendritic cells to induce a Th2 response, resulting in the production of Th2 cytokines, such as IL-4 and IL-13 [26]. TARC and TSLP are highly expressed in serum and AD lesions, leading to Th2-mediated allergic inflammation in the development of AD [27,28]. This study showed that the secretion levels of RANTES, TARC, and TSLP were dose-dependently reduced by NHGR treatment in TNF-α and IFN-γ-induced HaCaT cells. MAPK signaling pathways are significant mechanisms in inflammatory responses. It is reported that increased phosphorylation and activation of ERK, JNK, and p38 in AD mice and keratinocytes are a result of the inflammatory response in AD [29,30]. This study showed that the DfE-induced phosphorylation of ERK, JNK, and p38 was decreased by NHGR treatment. These results suggest that NHGR exerted anti-inflammatory activities by inhibiting the expression of TNF-α/IFN-γ-induced filaggrin, as well as TSLP, TARC, and RANTES via blockade of MAPK activation.

## 5. Conclusions

This study revealed that NHGR treatment ameliorates allergic skin inflammation by regulating immune dysfunction and skin barrier abnormality in a murine model in vitro. Our findings may lead to a promising new candidate for the treatment or prevention of AD.

## Figures and Tables

**Table 1:** Hematological parameters.

Group	WBC (×10^3^ Cells/μL)	WBC Differential Counting (%)					
		Neutrophil	Lymphocyte	Monocyte	Eosinophil	Basophil	Large Unstained Cell
NC	3.77 ± 0.47	22.24 ± 1.49	72.72 ± 1.59	0.70 ± 0.14	1.76 ± 0.21	0.26 ± 0.07	2.32 ± 0.40
DfE	2.39 ± 0.11 ^#^	39.85 ± 3.66 ^###^	60.24 ± 6.43	1.10 ± 0.18	2.72 ± 0.38 ^#^	0.46 ± 0.14	1.62 ± 0.34
NHGR200	2.43 ± 0.12	22.26 ± 2.71 ***	73.42 ± 2.63	0.78 ± 0.12	1.90 ± 0.13 *	0.48 ± 0.12	1.18 ± 0.32
NHGR400	2.98 ± 0.34	25.18 ± 0.90 **	69.78 ± 0.91	1.10 ± 0.19	1.48 ± 0.10 **	0.40 ± 0.04	2.06 ± 0.51
Dexa5	2.23 ± 0.13	36.38 ± 3.32	57.22 ± 3.74	1.42 ± 0.28	3.10 ± 0.39	0.38 ± 0.06	1.50 ± 0.38

WBC, white blood cells; NC, normal control; DfE, *Dermatophagoides farinae* extract; NHGR, *S. grosvenorii* residual extract (200 or 400 mg/kg); Dexa, dexamethasone (5 mg/kg). ^#^ *p* < 0.05 and ^###^ *p* < 0.001 indicate significant differences between the normal control and DfE-treated atopic dermatitis mice. * *p* < 0.05, ** *p* < 0.01, and *** *p* < 0.001 indicate significant differences between the DfE-treated atopic dermatitis mice and the NHGR- or Dexa-administrated group.
